# HIV drug resistance surveillance in low- and middle-income countries: 2004 to 2010

**DOI:** 10.7448/IAS.15.6.18083

**Published:** 2012-11-11

**Authors:** S Bertagnolio, N Parkin, M Jordan

**Affiliations:** 1 HIV Department, World Health Organization, Geneva, Switzerland; 2 Data First Consulting, Belmont, USA; 3 Division of Geographic Medicine and Infectious Disease, Department of Public Health and Community Medicine Tufts University School of Medicine, Boston, USA

## Abstract

**Background:**

At the end of 2011, over 8 million people were receiving antiretroviral therapy (ART) in low- and middle-income countries (LMIC), a 26-fold increase from 2003. Some degree of HIV drug resistance (HIVDR) will emerge among populations on combination ART even when high levels of adherence are achieved. In 2004, the World Health Organization (WHO) initiated global HIVDR surveillance to monitor emergence and transmission of HIVDR in countries scaling-up ART.

**Methods:**

WHO HIVDR surveillance strategy was designed to inform public health decision-making regarding choice of ART and to identify ART programme factors which could be adjusted to minimize HIVDR emergence. The strategy includes (1) surveillance of transmitted HIVDR (TDR) in recently infected populations, (2) surveillance of acquired HIVDR (ADR) in populations on ART and (3) monitoring of early warning indicators (EWI) of HIVDR which are ART programme factors favouring HIVDR emergence. Surveys used standardized protocols. Epidemiological and sequence data were quality assured.

**Results:**

*TDR*: Eighty-two surveys were conducted in 30 countries in 2004 to 2010, assessing 3588 recently infected individuals. Pooled analysis indicates an overall prevalence of 3.1% TDR to at least one drug class, 1.6% to non-nucleoside reverse transcriptase inhibitor (NNRTI), 1.3% to nucleoside reverse transcriptase inhibitor (NRTI) and 0.7% to protease inhibitor (PI). Levels of NNRTI resistance, particularly in the areas surveyed in Africa, increased over time, reaching 3.4% (95% CI=1.8 to 5.2%) in 2009. Greater ART coverage was associated, though modestly, with increased prevalence of TDR to NNRTI (*P*-value adjusted for region=0.039). *ADR*: Thirty-six ADR surveys assessing 6370 people in 12 LMIC were conducted in 2007 to 2010. HIVDR prevalence to any drug among those initiating ART ranged from 4.8% (95% CI=3.8 to 6.0%) in 2007 to 6.8% (95% CI=4.8 to 9.0%) in 2010. Ninety per cent of patients alive and on therapy at 12 months achieved viral load <1000 c/mL. Among people with virological failure, 72% had HIVDR to at least one drug. *EWI*: EWIs were monitored at 2017 clinics in 50 countries assessing 131,686 people since 2004. Overall, 75% of clinics met the target of 100% of patients receiving appropriate ART; 69% of clinics met the <20% target for lost to follow-up at 12 months; and only 65% of clinics provided a continuous supply of ART during a 12-month period.

**Conclusion:**

Expansion of ART in LMIC has resulted in an overall increase in HIVDR, particularly to NNRTI in Africa. EWIs reveal important gaps in service delivery and programme performance. While these data call for continued and improved scale up of surveillance, they also suggest that resistance is under control in the areas surveyed, and the majority of patients initiating or switching therapy are likely to respond to currently available first- and second-line therapy.

